# Conservative Treatment of Ankle Osteoarthritis

**DOI:** 10.3390/jcm10194561

**Published:** 2021-09-30

**Authors:** Sergio Tejero, Estefanía Prada-Chamorro, David González-Martín, Antonio García-Guirao, Ahmed Galhoum, Victor Valderrabano, Mario Herrera-Pérez

**Affiliations:** 1 Foot and Ankle Unit, Orthopedic Surgery and Traumatology Service, Hospital Universitario Virgen del Rocío, Av. Manuel Siurot, s/n, 41013 Sevilla, Spain; estefaniaprada@hotmail.com (E.P.-C.); aj_gargui90@hotmail.com (A.G.-G.); 2 School of Medicine (Health Sciences), University of Sevilla, Avda Dr. Fedriani, s/n, 41009 Sevilla, Spain; 3 Foot and Ankle Unit, Orthopedic Surgery and Traumatology Service, Hospital Universitario de Canarias, Carretera Ofra, s/n, 38320 Tenerife, Spain; davidglezmartin@gmail.com (D.G.-M.); herrera42@gmail.com (M.H.-P.); 4 School of Medicine (Health Sciences), Campus de Ofra, Universidad de La Laguna, San Cristóbal de La Laguna, s/n, 38071 Tenerife, Spain; 5 Orthopaedic and Trauma Department, Gerge Eliot Hospital, Nuneaton CV10 7DJ, UK; ahmed.galhoum@geh.nhs.uk; 6 Orthopaedic and Trauma Department, Swiss Ortho Center, Schmerzklinik Basel, Swiss Medical Network, Hirschg sslein 15, 4051 Basel, Switzerland; vvalderrabano@swissmedical.net

**Keywords:** conservative treatment ankle osteoarthritis, viscosupplementation, platelet-rich plasma, mesenchymal stem cells, biologics

## Abstract

Despite the disabling nature of ankle osteoarthritis (OA), there is poor scientific evidence for a conservative treatment compared to the hip and knee OA. In this regard, most of the treatment options in use are not based on clinical studies of the ankle, and they are extracted from evidence obtained from clinical studies of other lower limb joints. However, this does not seem to be a good idea, since the aetiology of ankle OA is quite different from that of the hip or knee. Nonpharmacological and pharmacological treatments such as nonsteroidal anti-inflammatory drugs, hyaluronic acid, corticosteroid, platelet-rich plasma injection and mesenchymal stem cells injections have been reported. However, further research is required in this field to obtain a specific clinical practice guideline for the conservative treatment of ankle OA.

## 1. Introduction

**Figure 1:**
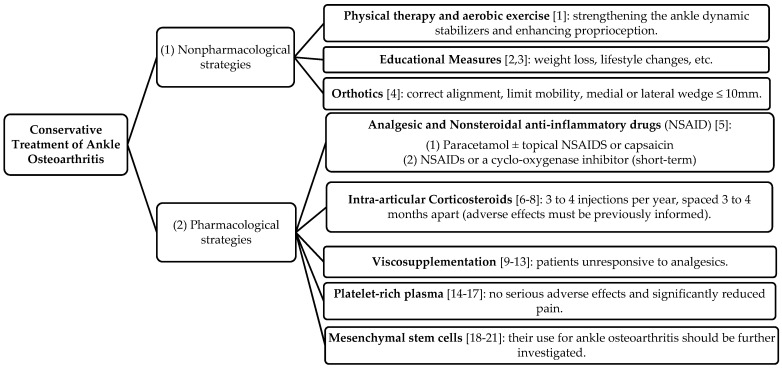
Conservative Treatment of Ankle Osteoarthritis: non-pharmacological (first step) and pharmacological (second step) strategies.

Although there are no clinical guidelines for the conservative management of ankle OA, the management recommendations for OA of the lower limb joints are used. However, unlike hip or knee OA patients, the ankle OA population encompasses a larger proportion of younger age groups. Those young patients require treatment options that will keep them very active yet delay the need for joint replacement or fusion surgery. Although several therapies exist, there is no consensus or guideline statement on a proper treatment algorithm for these patients. The majority of decisions are based on the treating surgeon’s experience and patient’s preferences. The nonpharmacological strategies are described in Section 2; however, many patients will experience symptoms that cannot be effectively controlled by these nonpharmacological measures; subsequently, pharmacological management (Section 3) or surgery will be needed. The aim of this narrative review is to provide a current concept regarding the conservative treatment options (Figure 1) for ankle osteoarthritis.

## 2. Nonpharmacological Strategies

### 2.1. Physical Therapy and Aerobic Exercise

It aims to improve function by strengthening the ankle dynamic stabilisers (calf, soleus, tibialis anterior and peroneal muscles), as well as enhancing the proprioception.

With OA progression, there is an early deterioration of the basic life activities, as well as a reduced ability to perform optimally at work. Occupational therapy and aerobic exercise are therefore essential, as they can improve energy conservation during walking and optimise the posture during work. Therapeutic modalities such as electrical stimulation, thermotherapy, electrotherapy or ultrasound can be used for symptomatic relief [1].

### 2.2. Educational Measures

A fundamental aspect of conservative treatment is patient education. It is crucial to explain to the patient the risk factors associated with OA and which of these risks can be modified. Obesity constitutes a risk factor for the onset and progression of variable musculoskeletal diseases. We must explain to the patient that, although there is little scientific evidence linking overweight to ankle osteoarthritis, weight loss has been shown to potentially reduce the pain [2]. A body mass index greater than twenty-five has a 1.5-fold increased risk for the diagnosis of OA [3]. Additionally, it is essential to modify lifestyles that promote OA, such as impact sports, going up and down stairs or slopes. The use of a cane for ambulation is recommended in case the patient needs it, since it can unload up to 25% of the body weight.

### 2.3. Orthotics

Orthotic treatment of the ankle joint OA has two objectives: (1) to reduce pain by maintaining correct alignment of the talus, which unloads the osteoarthritic parts of the joint surface, and (2) to limit the mobility of the ankle during walking in order to achieve mechanical unloading of the joint. An important aspect regarding the use of orthoses is patient compliance, since the large volume and stiffness of these orthoses interfere with long-term use [4]. One of the options for mechanical unloading is aligning the hindfoot by inserting a medial or a lateral wedge inside or outside the shoe; however, they have a limitation that they cannot exceed 10 mm. On the other hand, AFO (Ankle Foot Orthoses) can be particularly effective in patients with mechanical malalignment. 

## 3. Pharmacological Strategies

### 3.1. Analgesic and Nonsteroidal Anti-Inflammatory Drugs (NSAID)

Clinical practice guidelines for the other lower limb joints recommend paracetamol as the first line. It is reasonable to suggest that lower doses of 1000 mg/day may be administered initially and gradually increased if ineffective and if no side effects are encountered. Topical NSAIDS or capsaicin may be added. If symptoms are not controlled, NSAIDs or a cyclo-oxygenase inhibitor may be used to control the inflammation associated with the acute flare of ankle OA [5]. The efficacy of NSAIDs varies individually and decreases over time. Caution should be exercised with their long-term use due to the known side effects, especially in the older population.

### 3.2. Intra-Articular Corticosteroids

The use of corticosteroid injections is very useful as a diagnostic and therapeutic measure as it achieves immediate pain relief. The mechanism of action is based on its anti-inflammatory effect and reduction of leukocytes and lysosomal enzymes in the synovial fluid, although this may hurt the joint by decreasing the local immunity.

Most studies have shown a short duration effect, around 4–8 weeks, although a recent publication by Ward et al. reported benefits up to one year following a single injection [6]. They published the first prospective long-term follow-up study of patients treated with intra-articular corticosteroid injection of the ankle. They found a statistically significant improvement in the quality-of-life scores up to 6 months after the injection, although they did not find clinical changes associated with such improvements. They identified that improvement at 2 months post-injection can predict whether that beneficial effect could be sustained over a time up to 1 year post-injection or not [6]. The current recommendations advise against performing more than three or four injections per year, spaced 3 to 4 months apart, due to the harmful effects on articular cartilage [7]. Among the adverse effects are the local inflammatory reaction against the infiltrated material and depigmentation of the skin. It is extremely important to inform the patient beforehand, as this is a frequent cause of dissatisfaction [8].

### 3.3. Viscosupplementation

Hyaluronic acid is a component of synovial fluid and the extracellular matrix of the hyaline cartilage that is produced by chondrocytes and synoviocytes. The intra-articular injection of this substance is intended to restore the rheological properties of the synovial fluid. Its use in ankle osteoarthritis is usually tolerable and effective, with early clinical improvement in terms of pain, function, stiffness, quality of life, tolerability and reducing the need for analgesia. However, the role of viscosupplementation injections in the ankle joint, even of high molecular weight, remains controversial in the literature.

In a clinical trial, seventy-five patients with ankle osteoarthritis (OA) were randomised to receive either an intra-articular injection of botulinum toxin A (BoNT-A) or an intra-articular injection of hyaluronate with a home-based exercise program. The purpose of this study was to compare the efficacy of these two conservative treatment approaches concerning clinical and functional outcomes. The results at 6 months indicated that there were no significant differences between the groups concerning the Ankle Osteoarthritis Scale (AOS) scores, American Orthopedic Foot and Ankle Society (AOFAS) Ankle/Hindfoot scores, pain, single-leg stance test (SLS) scores, the Timed Up-and-Go test (TUG), patient global satisfaction, acetaminophen consumption and the incidence of adverse events [9]. On the other hand, in most of the studies, a significant improvement was observed up to 6 months from the injection. Some other trials have shown that these effects can last up to 12 and 18 months [10,11]. Cohen et al. conducted a double-blind randomised controlled trial (RCT) performing 5 weekly injections of 2cc of sodium hyaluronate and reported improvements in both the function and pain at 3 months [12]. However, a systematic review provided by the Cochrane database on 240 patients from six clinical trials on the use of hyaluronic acid in osteoarthritis of the ankle failed to draw definitive conclusions in favour of its standardised use. They recommend its conditional use in patients who do not respond to analgesics [13].

### 3.4. Platelet-Rich Plasma

Platelet-rich plasma injection is becoming very popular in orthopaedic surgery. It is a concentration of platelets extracted from autologous blood, which contains a high concentration of cytokines. These cytokines act by inducing cell proliferation and differentiation, in addition to promoting wound healing, especially transforming growth factor beta (TGF-B). This growth factor has antimicrobial properties and also contains a high amount of platelet-derived growth factors (PDGF) that help bone repair, prevention and treatment of soft tissue damage and the treatment of acute or chronic tendon injury.

Two studies of its application in the ankle are of particular note. Mei-dan et al. [14] compared the efficacy of hyaluronic acid and platelet-rich plasma in 30 patients with osteochondral lesions of the talus, with a follow-up of 28 weeks, evaluating the pain, stiffness and joint function using the VAS (Visual Analog Scale), AOFAS (American Orthopaedic Foot and Ankle Society) and AHFS (Ankle-Hind Foot Scale) scores. They concluded that, at 28 weeks, the patients who had platelet-rich plasma injections showed significantly less pain and better function. Angthong et al. [15] observed clinical improvement of the VAS scale with the application of ultrasound-guided or scoped platelet-rich plasma, with a mean follow-up of 16 months, although they did not observe radiological changes in the joint at 5 months of follow-up by MRI.

In vitro studies investigating the effects of platelet-rich plasma on chondrocytes demonstrated an increased proliferation rate, maintaining their marker expression, as well as stimulating matrix production and modulating inflammation [16]. An analgesic role is also suggested as a modulator of chondrocyte cannabinoid receptors, and it is thought that it may also enhance the synovial secretion of hyaluronic acid. A more recent study highlighted the efficacy and safety of this treatment on pain and physical function with a single injection only [17].

### 3.5. Mesenchymal Stem Cells

The chondrogenic differentiation capacity of adipocyte-derived stem cells (ASCs) is currently more accentuated, even greater than that of bone marrow-derived stem cells (BMSCs); however, their use for ankle osteoarthritis should be further investigated [18]. Their use in scaffolds, fibrin or hyaluronic acid appears to be more effective than isolated infiltration. The application of ASCs in combination with platelet-rich plasma to enhance their differentiation could improve the function and pain in various types of osteoarthritis of the lower limb joints, as has recently been published [19]. Emadedin et al. [20] evaluated a sample of 18 patients with OA at 2, 6, 12 and 30 months after the infiltration of bone marrow-derived mesenchymal stem cells and observed an increase in the walking distance in metres, as well as improved mean scores on the FAOS (Foot and Ankle Outcome Score) and WOMAC (Western Ontario and McMaster Universities Osteoarthritis Index) scales. They concluded that this treatment is safe and therapeutically beneficial, although only six ankle OA patients were included.

Recently, five randomised controlled trials were included in a meta-analysis that investigated the efficacy of mesenchymal stem cell injections in patients with knee osteoarthritis [21]. The included studies featuring various control groups, including a placebo injection, hyaluronic acid injection and no injection, as well various concomitant treatments, including high tibial osteotomy, microfracture, platelet-rich plasma injection and hyaluronic acid injection. There appeared to be a favourable outcome within 12–24 months after administration among patients who were administered intra-articular mesenchymal stem cells compared to patients who were administered either a control placebo or hyaluronic acid or no injection. Specifically, significant positive effects were observed in the analyses of VAS pain and Lysholm scores. Although there is no similar research in ankle OA, these results look promising. However, the posttraumatic aetiology of ankle OA is different from that of the knee.

## 4. Conclusions

Ankle osteoarthritis is a prevalent and disabling condition. However, very few studies investigating nonsurgical treatment options have been published. In general, education, exercise and weight loss are recommended. In the first instance, low doses of paracetamol in combination or not with topical nonsteroidal anti-inflammatory drugs (NSAIDs) or capsaicin may be used; however, if there is inadequate symptomatic relief, an oral NSAID or a cyclo-oxygenase-2 inhibitor could be added. On the other hand, orthobiological treatments have emerged as a growing area of innovation for osteoarthritis in recent years. In this sense, some studies have investigated intra-articular injections for ankle OA, and there is some evidence to suggest that hyaluronic acid or PRP may be effective in the short term for ankle OA. Additionally, positive effects were observed in limited cases through the intra-articular injection of mesenchymal stem cells, but no high-quality evidence has been reported. In conclusion, the relative efficacy of injectable orthobiological therapies is far from a definitive recommendation, and robust comparative trials are needed.

## Data Availability

Not applicable.
